# Assessment of Tp–e interval, Tp–e/QT, and Tp-e/QTc ratios in patients with acromegaly

**DOI:** 10.55730/1300-0144.5377

**Published:** 2022-03-02

**Authors:** İbrahim Etem ÇELİK, Sevde Nur FIRAT, Uğur BOZKURT, Mikail YARLIOĞLU, Mustafa DURAN, Sani Namık MURAT

**Affiliations:** 1 Department of Cardiology, Ankara Education and Research Hospital, University of Health Sciences Turkey, Ankara, Turkey; 2 Department of Endocrinology, Ankara Education and Research Hospital, University of Health Sciences Turkey, Ankara, Turkey

**Keywords:** Acromegaly, arrhythmogenesis, Tp-e interval

## Abstract

**Background/aim:**

Cardiovascular complications, including ventricular arrhythmias associated with abnormalities of ventricular repolarization, are the leading cause of morbidity and mortality in patients with acromegaly. Herein, we aimed to investigate ventricular repolarization using Tp–e interval, Tp-e interval/QT, and Tp-e interval/QTc ratios in acromegalic patients compared to healthy subjects.

**Materials and methods:**

A total of 29 patients (aged 51.9 ± 11.2, 65.5% women) with acromegaly and 30 control subjects (aged 47.3 ± 14.4, 63.3% women) were enrolled in the study. Tp–e and QT interval, corrected QT (QTc), Tp–e/QT, and Tp–e/QTc ratios were calculated from the 12-lead electrocardiogram.

**Results:**

Tp-e interval (89.28 ± 12.16 vs. 75.97 ± 9.92 ms; p < 0.001), Tp-e/QT ratio (0.237 ± 0.045 vs. 0.212 ± 0.029; p = 0.019), and Tp-e/QTc ratio (0.218 ± 0.031 vs. 0.195 ± 0.026; p = 0.003) were significantly higher in patients with acromegaly compared to control group. A positive correlation was determined between left atrial volume index (LAVI) and Tp-e interval (r = 0.272, p = 0.039).

**Conclusion:**

The current study is the first to have shown significantly increased Tp-e interval, Tp-e/QT ratio, and Tp-e/QTc ratio were increased in acromegalic patients. These results may be important for screening malignant arrhythmic events in acromegaly.

## 1. Introduction

Acromegaly is a rare disorder resulting from excessive growth hormone production (GH) and serum insulin like growth factor-1 (IGF-1) concentrations [1]. GH and IGF-1 excess are strongly linked to the development of cardiovascular complications including life-threatening arrhythmias, cardiomyopathy, hypertension, atherosclerosis, or valvular heart disease [2,3]. Cardiovascular complications are the most common causes of morbidity and mortality especially in patients with active acromegaly but frequently develop after adequate treatment in patients with the controlled disease [3–5]. The presence of cardiovascular comorbidities significantly increases the risk of morbidity and mortality in acromegalic patients than in the general population [3,6]. Malignant ventricular arrhythmia may develop in patients with acromegaly which is related to sudden cardiac death [7].

Ventricular repolarization abnormalities are central elements in arrhythmogenesis. Previous studies showed that ventricular repolarization can be evaluated by QT interval (QT), corrected QT interval (QTc), QT dispersion (QTd), and T wave measurements [8,9]. The time between the peak and the end of the T wave (Tp-e interval) is a measure of transmural dispersion of repolarization time and seems to play a major role in arrhythmogenesis [10,11]. Increased Tp-e interval, Tp-e interval/QT, and Tp-e interval/QTc ratios have been accepted as electrocardiographic markers to predict ventricular arrhythmias and cardiovascular mortality [12,13]. However, the Tp-e/QT ratio has been emphasized among the more accurate marker of ventricular repolarization [13,14].

The aim of the present study was to assess ventricular repolarization parameters of acromegalic patients using Tp-e interval, Tp-e interval/QT, and Tp-e interval/QTc ratios. To our knowledge, no prior studies have examined previously the association between these parameters and acromegaly.

## 2. Methods

### 2.1. Study population

In this study, we enrolled consecutively eligible outpatients of the Endocrinology Department with the diagnosis of acromegaly. A total of 33 acromegalic patients and 30 healthy individuals were enrolled in the study, 4 acromegalic patients were excluded. Patients with renal dysfunction (n = 2), acute or chronic infection, cardiovascular diseases including ischaemic heart disease (n = 1), congestive heart failure, severe valvular heart disease, abnormal serum electrolyte values (n = 1), pregnancy, malignancy, left and right bundle branch block, Mobitz type II, third-degree atrioventricular block, ventricular preexcitation, atrial fibrillation, a permanent pace-maker, severe kidney, liver and lung disease, those taking any class I and III antiarrhythmic medication were excluded from the study. Therefore, 29 patients with acromegaly and 30 gender and age-matched healthy volunteers were included in the study after exclusion criteria were applied.

### 2.2. Electrocardiography

The 12-lead electrocardiogram (ECG) was recorded at a paper speed of 50 mm/s (Mortara, Milwaukee, USA) at rest in the supine position. All of the ECGs were scanned and then Adobe Photoshop software was used for ×400% magnification. ECG measurements of QT and Tp-e intervals were performed by two cardiologists who did not know the patient data. The Tp-e interval was defined as the difference between the peak of the T wave and the end of the T wave. The U wave was excluded from the Tp-e interval. The QT interval was measured from the beginning of the QRS complex to the end of the T wave and Corrected QT (QTc) was calculated with the Bazett formula: cQT = QT√(R−R interval). Measurements of the Tp-e interval were evaluated from precordial leads. The Tp-e/QT ratio was calculated from these measurements. Additionally, twenty-four hour holter recordings were evaluated by using three-channel analog recorders using the ELATEC Holter system.

### 2.3. Echocardiography

The echocardiographic assessment was analysed by using a Vivid S60N Dimension Cardiovascular Ultrasound System (Vingmed-General Electric, Horten, Norway) with a 3.5 MHz transducer in the left lateral decubitus position. Parasternal long and short-axis views and apical views were used as standard imaging windows. The ejection fraction was calculated by using the modified Simpson method. Septal and posterior walls’ thickness, left ventricular (LV) end-systolic and end-diastolic dimensions left atrial diameter were measured from parasternal long-axis view. Tissue Doppler imaging (TDI) was acquired from the lateral and septal walls of the left ventricular basal section. E/A ratio and E/e′ ratios were analysed in the evaluation of LV diastolic function. Left ventricular mass index (LVMI) was calculated by the ratio of calculated LV mass to the body surface. Left ventricular hypertrophy (LVH) was defined as the calculation of LVMI > 115 g/m2 in men and >95 g/m2 in women. Left atrial (LA) volume was assessed by the biplane area-length method from apical 4- and 2-chamber views. LA volume/body surface area was measured for LA volume index (LAVI). All these echocardiographic measurements were performed by experienced investigators according to standards outlined by the American Society of Echocardiography guidelines [15].

### 2.4. Laboratory evaluation

Serum GH and IGF-1 levels were measured by using cobas 8000 Modular Analyzer Series (Roche Diagnostics, Mannheim, Germany). Biochemical measurements including fasting blood glucose, total cholesterol, low-density lipoprotein (LDL) cholesterol, high-density lipoprotein (HDL) cholesterol, triglyceride, serum creatinine, serum electrolytes were evaluated using cobas 8000 Modular Analyzer Series (Roche Diagnostics, Mannheim, Germany) (Abbott Aeroset, USA). Complete blood count (CBC) was calculated by an automated analyzer (Sysmex SF-3000 Hematology Analyzer, USA).

### 2.5. Statistical analysis

All data analyses were performed with SPSS version 22.0 (SPSS, Inc, Chicago, Illinois). Categorical variables are expressed as counts and percentages, and continuous variables are presented as mean ± standard deviation or as median and interquartile ranges. Categorical variables were compared by the chi-square test or Fisher’s exact tests as appropriate. Comparisons of parametric values between the 2 groups were compared using independent samples t-test. Comparisons of nonparametric values between the 2 groups were done by Mann-Whitney U test. The goodness of fit of the model was evaluated by the Hosmer-Lemeshow test. Pearson correlation analysis was performed to examine the relationship between Tp−e interval, Tp−e/QT ratio, and Tp−e/QTc and other variations. A 2-sided p-value of < 0.05 was considered statistically significant.

## 3. Results

The baseline clinical characteristics and laboratory parameters of the patients are shown in Table 1. The mean age of the subjects was 51.9 ± 11.2 years, and 19 (65.5%) of the patients were female. There was no statistically significant difference between the groups in terms of age, gender, hypertension, diabetes mellitus, hyperlipidemia, smoking habitus, and basal laboratory findings (p > 0.05). However, body mass index (BMI) was higher in the acromegaly group (p < 0.001). Also, serum GH (1.39 (3.37–0.55) vs. 0.32 (0.78–0.09); p < 0.001) and IGF-1 (249.9 ± 189.3 ng/mL vs. 147.1 ± 39.6 ng/m; p = 0.009) levels were significantly higher in patients with acromegaly.

**Figure 1:**
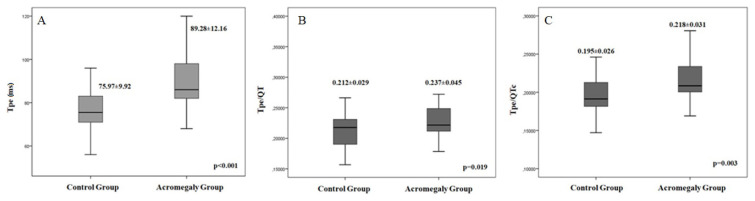
Comparison of Tp-e Interval, Tp-e/QT, and Tp-e/QTc ratios between acromegalic patients and healthy control subjects.

The electrocardiographic and echocardiographic findings are given in Table 2. The mean heart rate (78.4 ± 9.8 vs. 76.9 ± 14.4 per minute; p = 0.638) was similar between the groups. QRS duration (94.3 ± 13.1 vs. 86.2 ± 8.3; p = 0.007), QT (380.2 ± 28.8 vs. 357.5 ± 17.3 ms; p = 0.001), and QTc intervals (409.6 ± 15.1 vs. 389.5 ± 14.0; p < 0.001) were determined to be statistically significantly higher in the acromegaly group. Additionally, Tp-e interval (89.28 ± 12.16 vs. 75.97 ± 9.92 ms; p < 0.001), Tp-e/QT (0.237 ± 0.045 vs. 0.212 ± 0.029; p = 0.019), and Tp-e/QTc ratios (0.218 ± 0.031 vs. 0.195 ± 0.026; p = 0.003) were also significantly increased in patients with acromegaly compared to control group (Figure 1). We had 24 h holter recordings of study groups and 3 of 29 acromegalic patients had non sustained ventricular tachyarrhythmias (NSVT). Acromegaly patient with NSVT attack had similar QRS duration (112 ± 10.1 vs. 90.1 ± 10.3; p = 0.062), QT (402 ± 25.2 vs. 366.9 ± 24.9 ms; p = 0.067), and QTc intervals (405,4 ± 15.6 vs. 397.7 ± 16; p = 0.075), Tp-e interval (82 ± 8.7 vs. 80.5 ± 10.1 ms; p = 0.660), Tp-e/QT (0.203 ± 0.011 vs. 0.220 ± 0.028; p = 0.147), and Tp-e/QTc ratios (0.192 ± 0.019 vs. 0.202 ± 0.026; p = 0.523) with acromegaly patient without NSVT attack.

There were no significant differences between the two groups in the echocardiographic dimensions including the left ventricular ejection fraction, left ventricle end-diastolic diameter and left ventricle end-sistolic diameter (p > 0.05). Acromegalic patients exhibited significantly higher values in interventricular septum (9.9 ± 1.5 vs. 8.8 ± 1.4; p = 0.005), left ventricular posterior wall thickness (9.5 ± 1.3 vs. 8.7 ± 1.4; p = 0.032), LVMI (90.8 ± 24.9 vs. 74.6 ± 15.0; p = 0.004) and left atrial diameter (36.4 ± 4.3 vs. 29.8 ± 4.6; p < 0.001) compared to control group. Similarly, LA volume (45.9 ± 17.0 vs. 34.1 ± 9.4; p = 0.002), LA area (16.9 ± 3.9 vs. 13.2 ± 2.5; p < 0.001) and LAVI (23.3 ± 7.7 vs. 18.6 ± 3.8; p = 0.006) were also significantly higher in patients with acromegaly. TDI demonstrated e’ lateral value (11.1 ± 2.7 vs. 13.5 ± 2.9; p = 0.02) was significantly lower in acromegalic patients. Additionally, no significant difference was observed between E/A and E/e′ values of patients between the groups (p > 0.05).

**Figure 2:**
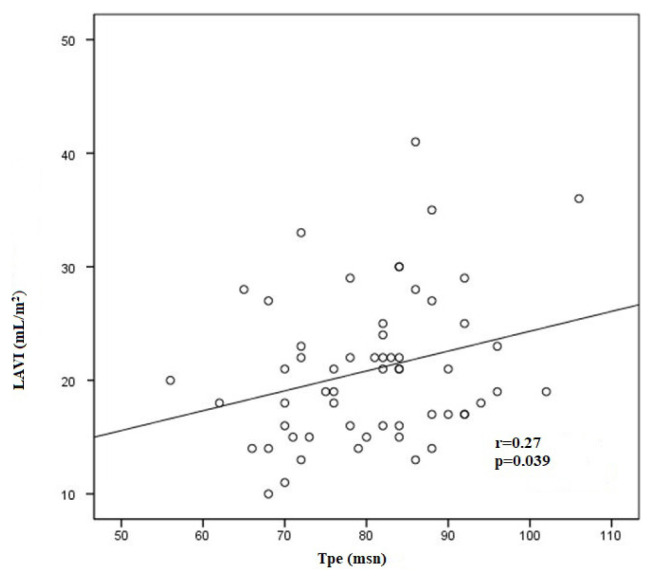
Correlation between Tp-e interval and left atrium volume index (LAVI).

Finally, the Pearson correlation coefficient was performed to estimate the correlation between LAVI and Tp-e interval. We found a significant positive correlation between LAVI and Tp-e interval (r = 0.272, p = 0.039; Figure 2).

## 4. Discussion

In this study, we evaluated ventricular repolarization parameters in patients with acromegaly and compared them with healthy control subjects. Our study results indicate that acromegalic patients had longer Tp-e interval, Tp-e/QT, and Tp-e/QTc ratios when compared to healthy subjects. To the best of our knowledge, this study was the first clinical trial focusing on the Tp−e interval, Tp−e/QT, Tp−e/QTc ratios in acromegalic patients.

Cardiovascular diseases are one of the most prevalent in acromegalic patients and in 60% of uncontrolled acromegalic patients die from cardiovascular comorbidities [16,17]. In this regard, hypertension, cardiomyopathy, heart valve disease, atherosclerosis, and coronary artery disease are important manifestations of cardiac involvement in patients with acromegaly [3,18]. Cardiac rhythm abnormalities are also frequent and ectopic beats, bundle branch block, paroxysmal atrial fibrillation, paroxysmal supraventricular tachycardia, sick sinus syndrome, and ventricular tachycardia have been demonstrated [18,19]. The underlying pathophysiological mechanisms of each cardiac involvement may not be fully understood but persistent GH and IGF-I excess, age, prolonged disease duration, and coexistence of other cardiovascular risk factors may play an important role as predisposing factors [18]. GH and IGF-1 excess are strongly related to the development of cardiovascular complications, especially in patients with active acromegaly but frequently persist after adequate treatment in patients with the controlled disease [3,4]. GH and IGF-1 have various negative effects on the cardiovascular system like microvascular inflammation, oxidative stress, and endothelial dysfunction which may explain the development of cardiovascular complications [4].

Ventricular repolarization parameters are related to malignant arrhythmias and have prognostic significance in terms of mortality and sudden cardiac death. Several studies have shown that QT, QTc, QTd, and T wave measurements were clarified as a sign of increased dispersion of ventricular repolarization [8,9]. Tp-e interval which is a measure of transmural dispersion of repolarization time seems to play a major role in arrhythmogenesis [10,11]. Increased Tp-e interval, Tp-e interval/QT, and Tp-e interval/QTc ratios have been evaluated as electrocardiographic markers of ventricular arrhythmias and cardiovascular mortality [12,13]. Due to the interaction with the QT and heart rate and body weight, Tp-e/QTc ratio has been emphasized as a better indicator of ventricular repolarization [10,13]. Prolonged Tp-e interval was associated with increased mortality in QT syndrome and Brugada syndrome [12,13]. In recent years, Tp-e interval, Tp-e interval/QT, and Tp-e interval/QTc ratios have been investigated in many diseases. Yenercağ et al. [20] demonstrated that Tp-e interval, Tp-e/QT, and Tp-e/QTc ratios were significantly increased in Fabry patients. Another study showed that Tpe, Tpe/QT, and Tpe/QTc ratios were higher in acute myocarditis patients [21]. Moreover, Tp-e interval, Tp-e/QT ratio, and Tp-e/QTc ratio were increased in asymptomatic arrhythmogenic right ventricular cardiomyopathy patients [22].

Malignant ventricular arrhythmias and sudden death can be seen higher in acromegalic patients compared to the general population [23]. The severity of ventricular arrhythmias was associated with the duration of disease and correlated with left ventricular mass [19]. The main factor involved in the onset and progression of arrhythmia in acromegaly is thought to be myocardial interstitial fibrous due to GH and IGF-1 excess [24]. The determination of clinical predictors of ventricular arrhythmias is important in acromegalic patients. Previous studies have evaluated QT intervals in patients with acromegaly to identify patients at high risk for sudden death and prolonged QT duration was observed in acromegaly [23,25]. Dural et al. [26] also investigated cardiac autonomic dysfunction for early identification of acromegalic patients at higher risk. Additionally, the occurrence of late potentials, which is an indicator of the risk of ventricular arrhythmia, was higher in patients with acromegaly [27]. Consistent with previous reports, in our study, we observed prolonged QT intervals. However, noninvasive and easy measurable ECG parameters, such as Tp-e interval, Tpe/QT, and Tpe/QTc ratios have not been investigated in patients with acromegaly before. In the current study, Tp-e interval, Tp-e/QT, and Tp-e/QTc ratios were significantly higher in acromegalic patients than in control subjects.

The relationship between diastolic dysfunction and Tp-e interval, Tp-e/QT, and Tp-e/QTc ratios which may be related to cardiomyocyte involvement and increased risk of arrhythmia has been shown in Fabry patients [20]. Regarding the evaluation of left ventricular diastolic dysfunction in acromegaly, Cansu et al. [28] investigated diastolic dysfunction parameters in acromegalic patients compared to normal individuals and found that LAVI, a diagnostic criterion for diastolic dysfunction, was significantly higher in the patients with acromegaly. In addition, it was reported that LAVI was a potential predictor of arrhythmias and adverse outcomes in patients with hypertrophic cardiomyopathy including sudden cardiac death [29]. In this regard, we analyzed the relationship between diastolic dysfunction and ventricular arrhythmia parameters in our study. A significant positive correlation was detected between the LAVI and Tp-e interval in the study cohort. Thus, diastolic dysfunction in acromegalic patients may be associated with a higher risk of arrhythmia.

The results of the current study should be assessed with some limitations. First, this study is a single-center and relatively small sample population. Second, this is a cross-sectional study so we could not follow the arrhythmic episodes of patients. Finally, we did not have the data demonstrating at what time point when the Tp-e interval, Tp-e/QT, and Tp-e/QTc ratios started to prolong or change following diagnosis of acromegaly. Future large, prospective and randomized clinical trials are required to assess the clinical applicability of our findings.

## 5. Conclusion

In conclusion, Tp-e interval, Tp-e/QT, and Tp-e/QTc ratios were increased in patients with acromegaly, which may indicate a higher risk for ventricular arrhythmias in patients with acromegaly. Tp-e interval, Tp-e/QT, and Tp-e/QTc ratios are simple, inexpensive, and noninvasive methods that may be valuable for screening for ventricular repolarization in acromegalic patients.

## Figures and Tables

**Table 1:** Baseline characteristics and laboratory parameters of the study subjects.

	Control group (n = 30)	Acromegaly (n = 29)	p value
Age (years)	47.3 ± 14.4	51.9 ± 11.2	0.183
Women, n (%)	19 (63.3%)	19 (65.5%)	0.861
Body mass index (kg/m^2^)	25.5 ± 4.5	30.5 ± 5.7	<0.001
Hypertension, n (%)	5 (16.6%)	11 (37.9%)	0.066
Diabetes mellitus, n (%)	10 (33.3%)	12 (41.3%)	0.523
Current smoker, n (%)	9 (30.0%)	10 (31.0%)	0.931
Hypercholesterolemia, n (%)	4 (13.3%)	11 (37.9%)	0.066
Hemoglobin (g/dL)	13.8 ± 1.5	13.1 ± 1.2	0.079
Serum creatinine (mg/dL)	0.71 ± 0.14	0,73 ± 0.17	0.712
Sodium (mEq/L)	14.1 ± 3.2	140.6 ± 3.1	0.556
Potassium (mmol/L)	4.4 ± 0.2	4.4 ± 0.2	0.979
Calcium (mg/dL)	9.5 ± 0.4	9.6 ± 0.7	0.428
Magnesium (mmol/L)	0.81 ± 0.06	0.78 ± 0.08	0.117
Total cholesterol (mg/dL)	175.0 ± 31.1	171.6 ± 43.1	0.756
High density lipoprotein cholesterol (mg/dL)	51.9 ± 14.7	48.9 ± 12.1	0.431
Low density lipoprotein cholesterol (mg/dL)	99.6 ± 26.2	90.9 ± 38.5	0.357
Triglyceride (mg/dL)	99 (158–70)	111.0 (211.2–83.0)	0.092
GH (ng/mL)	0.32 (0.78–0.09)	1.39 (3.37–0.55)	<0.001
GF-1 (ng/mL)	147.1 ± 39.6	249.9 ± 189.3	0.009

Data are given as mean ± standard deviation, median (interquantile range) or n (%). GH growth hormone, IGF insulin-like growth factor.

**Table 2:** Electrocardiographic and echocardiographic findings of the study population.

	Control group (n = 30)	Acromegaly (n = 29)	p value
Heart rate (bpm)	78.4 ± 9.8	76.9 ± 14.4	0.638
Tp−e interval (ms)	75.97 ± 9.92	89.28 ± 12.16	<0.001
QT interval (ms)	357.5 ± 17.3	380.2 ± 28.8	0.001
QTc interval (ms)	389.5 ± 14.0	409.6 ± 15.1	<0.001
QRS interval (ms)	86.2 ± 8.3	94.3 ± 13.1	0.007
Tp-e/QT ratio	0.212 ± 0.029	0.237 ± 0.045	0.019
Tp−e/QTc ratio	0.195 ± 0.026	0.218 ± 0.031	0.003
LVEF (%)	63.6 ± 4.9	62.4 ± 5.4	0.414
LVEDD (mm)	46.4 ± 5.2	48.8 ± 4.5	0.067
LVESD (mm)	30.3 ± 4.3	31.7 ± 4.0	0.206
IVS (mm)	8.8 ± 1.4	9.9 ± 1.5	0.005
PW (mm)	8.7 ± 1.4	9.5 ± 1.3	0.032
LVMI (g/m2)	74.6 ± 15.0	90.8 ± 24.9	0.004
Left atrial diameter (mm)	29.8 ± 4.6	36.4 ± 4.3	<0.001
LAa (cm2)	13.2 ± 2.5	16.9 ± 3.9	<0.001
LAv (cm3)	34.1 ± 9.4	45.9 ± 17.0	0.002
LAVI (mL/m2)	18.6 ± 3.8	23.3 ± 7.7	0.006
E/A ratio	1.2 ± 0.3	1.0 ± 0.3	0.051
e′ lateral (cm/s)	13.5 ± 2.9	11.1 ± 2.7	0.003
E/e′ ratio	5.5 ± 1.2	6.4 ± 2.4	0.116

Data are represented as mean values ± SD or (%). LVEF Left ventricular ejection fraction, LVEDD Left ventricle end-diastolic diameter, LVESD Left ventricle end-systolic diameter, LAa Left atrial area, LAv Left atrial volume, LAVI left atrial volume index, IVS Interventricular septum thickness, PW Posterior wall diameter, LVMI Left ventricular mass index mm millimeters, bpm beats per minute, ms milliseconds.
